# Supplementary material for: Site-specific MCM sumoylation prevents genome rearrangements by controlling origin-bound MCM
Source: PLoS Genet. 2022 Jun 13;18(6):e1010275. doi: 10.1371/journal.pgen.1010275 (PMC9232163; doi:10.1371/journal.pgen.1010275)
Supplement: S2 Table — (DOCX) [file pgen.1010275.s004.docx]

**S2 Table. Yeast strains used.**

| HZY2101 | MATa, *HF-SMT3*, *sml1Δ:TRP1, arg4Δ, ura3–52, leu2Δ1, trp1Δ63, his3Δ200, lys2ΔBgl, hom3–10, ade2Δ, ade8* | Albuquerque et al., 2013 [1] |
| --- | --- | --- |
| HZY4172 | MATa, *mcm3Δ::NAT*, pRS316-*MCM3*, *HF-SMT3*, derived from HZY2101 | This study |
| HZY4173 | MATalpha, *mcm3Δ::NAT*, pRS316-*MCM3*, *HF-SMT3*, derived from HZY2101 | This study |
| HZY4184 | MATa, *mcm3-29KR-1(K767R)::HIS3*, *HF-SMT3*, derived from HZY4172 | This study |
| HZY4177 | MATalpha, *mcm3-29KR(K767R)-1::HIS3*, *HF-SMT3*, derived from HZY4173 | This study |
| HZY4215 | MATalpha, *mcm3-29KR(K767R)-1::HIS3, mms21-CH::KanMX*, *HF-SMT3*, derived from HZY4177 | This study |
| HZY4221 | MATa, *mcm3-29KR(K767R)-1::HIS3, siz1Δ:: KanMX, siz2Δ::URA3*, *HF-SMT3*, derived from HZY4184 | This study |
| HZY4189 | MATa, *mcm3-29KR(K767R)-1::HIS3, ulp2Δ::KanMX*, *HF-SMT3*, derived from HZY4184 | This study |
| HZY4219 | MATa, *mcm3-29KR(K767R)-1::HIS3, bar1Δ::URA3*, *HF-SMT3*, derived from HZY4184 | This study |
| HZY3916 | MATa, *cdc6-1*:: *LEU2,* *mcm3-29KR(K767R)::HIS3, bar1Δ::URA3*, *HF-SMT3*, derived from HZY4184 | This study |
| HZY2309 | S288C MATa, *mcm3-K767R::HIS3*, *HF-SMT3*, *sml1Δ::TRP1, arg4Δ, ura3–52, leu2Δ1, trp1Δ63, his3Δ200, lys2ΔBgl, hom3–10, ade2Δ, ade8,* derived from HZY2101 | This study |
| HZY2262 | S288C MATa, *mcm3-K768R::HIS3, HF-SMT3*, *sml1Δ::TRP1, arg4Δ, ura3–52, leu2Δ1, trp1Δ63, his3Δ200, lys2ΔBgl, hom­3–10, ade2Δ, ade8,* derived from HZY2101 | This study |
| HZY1954 | S288C MATa, *mcm3-2KR::HIS3*, *bar1Δ::URA3*, *sml1Δ::TRP1, HF-SMT3*, *arg4Δ, ura3–52, leu2Δ1, trp1Δ63, his3Δ200, lys2ΔBgl, hom3–10, ade2Δ, ade8,* derived from HZY2101 | This study |
| HZY1833 | S288C MATa, *PSF2-TAF::KanMX bar1Δ::URA3*, *sml1Δ::TRP1,* *arg4Δ, ura3–52, leu2Δ1, trp1Δ63, his3Δ200, lys2ΔBgl, hom3–10, ade2Δ, ade8* | This study |
| HZY1957 | S288C MATa, *PSF2-TAF::KanMX mcm3-2KR::HIS3 bar1Δ::URA3*, *sml1Δ::TRP1,* *arg4Δ, ura3–52, leu2Δ1, trp1Δ63, his3Δ200, lys2ΔBgl, hom3–10, ade2Δ, ade8* | This study |
| HZY1959 | S288C MATa, *mcm3-2KR:HIS3*, *bar1Δ::URA3*, *sml1Δ::TRP1, arg4Δ, ura3–52, leu2Δ1, trp1Δ63, his3Δ200, lys2ΔBgl, hom3–10, ade2Δ, ade8* | This study |
| HZY1077 | W303 MATa, *ade2-1*, *can1-100*, *his3-1115*, *leu2-3,112*, *trp1-1*, *ura3-1*, *RAD5+* | This study |
| HZY234 | W303 MATa, *mcm3Δ::NAT*, pRS316-*MCM3*, derived from HZY1077 | This study |
| HZY549 | S288C MATa, *ura3–52, leu2Δ1, trp1Δ63, his3Δ200, lys2ΔBgl, hom3–10, ade2Δ, ade8, sml1Δ::TRP1, arg4Δ*, ade8 has 2 NT loss | This study |
| HZY4237 | MATa, *Hyg:: 6xHIS-3xHA-MCM3 bar1Δ::HIS sml1Δ::TRP* | This study |
| HZY4270 | Diploid, *Hyg::6xHIS-3xHA-MCM3/MCM3, BAR1/bar1Δ::URA3* | This study |
| HZY1970 | Diploid, *Hyg::3xFLAG-Mcm3/URA3:6HIS-3HA-Mcm3, SML1/sml1Δ:TRP* | This study |
| HZY2233 | Diploid, *Hyg::3xFLAG-MCM3/Hyg::6HIS-3HA-mcm3-2KR::HIS3*, *BAR1/bar1Δ::URA3* | This study |
| HZY2257 | Diploid, *URA::6HIS-3HA-MCM3/Hyg::3xFLAG-mcm3-2KR::HIS3* | This study |
| HZY2220 | Doploid, *KanMX::3xFLAG-mcm3-2KR::HIS3/Hyg::6HIS-3HA-mcm3-2KR:HIS3* | This study |
| yJF38 | W303 MATa, *pep4:KanMX4* *bar1Δ::hph-NT1*, *his3-11::HIS3*pJF2 (GAL4-GAL1-10-*CDT1*) *trp1-1::TRP*1pJF3 (*MCM5*-GAL1-10-*MCM4*) *leu2-3::LEU2*pJF4 (*MCM7*-GAL1-10-*MCM6*) *ura3-1::URA3* pJF6 (*MCM2*-GAL1-10-3xFLAG-*MCM3*) | Frigola et al., 2013 [2] |
| HZY2301 | W303 MATa, *pep4:KanMX4* *bar1Δ::hph-NT1*, *his3-11::HIS3*pJF2 (GAL4-GAL1-10-*CDT1*) *trp1-1::TRP*1pJF3 (*MCM5*-GAL1-10-*MCM4*) *leu2-3::LEU2*pJF4 (*MCM7*-GAL1-10-*MCM6*) *ura3-1::URA* p(*MCM2*-GAL1-10-3xFLAG-*mcm3-2KR*) | This study |
| yJF2966 | MATa, *TRP1::*GAL1-10-*ORC5*-*ORC6*, *HIS3*::GAL1-10-*ORC3*-*ORC4*, *URA3*::GAL1-10-*ORC1*-*ORC2*, *ade2-1,* *ura3-1, his3-11,15 trp1-1 leu2-3,112 can1-100, bar1::hyg, pep4::kanMX6* | Frigola et al., 2013 [2] |
| HZY2131 | MATa, dGCR assay, also known as RDKY6678 | Putnam et al., 2009 [3] |
| HZY2337 | MATa, *mcm3-K767R/K768R::HIS3,* dGCR assay | This study |
| HZY2338 | MATalpha, *mcm3-K767R/K768R::HIS3,* dGCR assay | This study |
| HZY2308 | MATa, *mcm3-K767R::HIS3,* dGCR assay | This study |
| HZY2341 | MATa, *mcm3-K768R::HIS3,* dGCR assay | This study |
| HZY2342 | MATalpha, *mcm3-K768R::HIS3,* dGCR assay | This study |

**Reference:**

1. Albuquerque CP, Wang G, Lee NS, Kolodner RD, Putnam CD, Zhou H. Distinct SUMO ligases cooperate with Esc2 and Slx5 to suppress duplication-mediated genome rearrangements. PLoS genetics. 2013;9(8):e1003670.

2. Frigola J, Remus D, Mehanna A, Diffley JF. ATPase-dependent quality control of DNA replication origin licensing. Nature. 2013;495(7441):339-43.

3. Putnam CD, Hayes TK, Kolodner RD. Specific pathways prevent duplication-mediated genome rearrangements. Nature. 2009;460(7258):984-9.
