# Supplementary material for: Site-specific MCM sumoylation prevents genome rearrangements by controlling origin-bound MCM
Source: PLoS Genet. 2022 Jun 13;18(6):e1010275. doi: 10.1371/journal.pgen.1010275 (PMC9232163; doi:10.1371/journal.pgen.1010275)
Supplement: S3 Table — (DOCX) [file pgen.1010275.s005.docx]

**S3 Table. Plasmids used.**

| HZE1997 | pRS316-*MCM3* | This study |
| --- | --- | --- |
| HZE2019 | pRS315-*MCM3* | This study |
| HZE3004 | pRS316-*3xFLAG-MCM3* | This study |
| HZE2973 | pRS315-*mcm3-39KR::HIS3* | This study |
| HZE2970 | pRS315-*mcm3-36KR::HIS3* | This study |
| HZE2977 | pRS315-*mcm3-33KR::HIS3* | This study |
| HZE2320 | pRS315-*mcm3-30KR::HIS3* | This study |
| HZE2308 | pRS315-*mcm3-19KR::HIS3* | This study |
| HZE2976 | pRS315-*mcm3-15KR::HIS3* | This study |
| HZE2307 | pRS315-*mcm3-8KR::HIS3* | This study |
| HZE2160 | pRS315-*6HIS-3HA-mcm3-K767R* | This study |
| HZE2161 | pRS315-*6HIS-3HA-mcm3-K768R* | This study |
| HZE2241 | pRS315-*mcm3-K767/768R* (2KR) | This study |
| HZE2287 | pRS315-*mcm3-7KR-1*::*HIS3* (K767R) | This study |
| HZE2288 | pRS315-*mcm3-7KR-2::HIS3* (K768R) | This study |
| HZE2971 | pRS315-*mcm3-38KR-1::HIS3* (K767R) | This study |
| HZE2972 | pRS315-*mcm3-38KR-2::HIS3* (K768R) | This study |
| HZE2247 | pRS315-*mcm3-4KR::HIS3* | This study |
| HZE2236 | pRS315-*mcm3-7KR::HIS3* | This study |
| HZE2261 | pRS315-*mcm3-14KR-1::HIS3* (K767R) | This study |
| HZE2258 | pRS315-*mcm3-14KR-2::HIS3* (K768R) | This study |
| HZE2255 | pRS315-*mcm3-29KR-1::HIS3* (K767R) | This study |
| HZE2256 | pRS315-*mcm3-29KR-2::HIS3* (K768R) | This study |
| HZE1134 | pGEX-6P1-*CDC6* | This study |
| HZY3004 | pRS315-*3xFLAG-MCM3* | This study |
| HZY3081 | pRS315-*3xFLAG-mcm3-2KR* | This study |
