# Supplementary material for: Site-specific MCM sumoylation prevents genome rearrangements by controlling origin-bound MCM
Source: PLoS Genet. 2022 Jun 13;18(6):e1010275. doi: 10.1371/journal.pgen.1010275 (PMC9232163; doi:10.1371/journal.pgen.1010275)
Supplement: Attachment — Submitted filename: Responses to Reviewers.docx [file pgen.1010275.s006.docx]

**Responses to Reviewer 1.**

1) Figure 4A, B and C require quantitation. Is the effect reproducible and significant? N=3 is required.

> See revised Figure 4 and new Supplementary Figure 2. New co-IP experiment has been performed, showing that *mcm3-2KR* reduces the amount of loaded MCM in G1 but not MCM complex formation in G2/M when loaded MCM is absent. Because this experiment does not distinguish single versus double hexamer, their relative amounts are unknown. Considering MCM single hexamer is an intermediate *en route* to become double hexamer, the defect of *mcm3-2KR* could affect one or more steps during MCM loading, which has yet to be determined. Collectively, these results demonstrate that *mcm3-2KR* reduces the amount of loaded MCM in cells. Our initial experiment shows that *mcm3-2KR* has no detectable effect on the *in vitro* MCM loading, and this direction requires further studies.

2) Figure 5B, C require quantitation. Are the effects significant and reproducible? Fig. 5 B and C make the same point, which may obviate the need for n=3.

> See revised Figure 5 with quantification. These results show that the CMG level is consistently lower in *mcm3-2KR* cells, in complete agreement with the lower amount of loaded MCM (Figures 3 and 4). This defect is also consistent with slower cell cycle progression and GCR accumulation, suggesting that the MCM and CMG levels are the rate-limiting factors of eukaryotic DNA replication.

3) Figure 6: K768R and 2KR have nearly the same increase in dGCR, but only 3KR shows growth delay. Why? Some discussion should be added.

> *mcm3-2KR* has a modestly higher rate (~2-fold) of dGCRs than that of *mcm3-K768R* and a severe growth defect (2-fold slower), which is more readily observed. However, we caution against making a quantitative correlation between cell growth and GCR rate, which are vastly different. For instance, the GCR assay measures specific events near the telomere on the left arm of Chromosome-V, while cell growth is a collective response of all origin activities. We have included a brief discussion about this (Line 190-196).

4) The authors hypothesize that incomplete replication is the cause for the increase in dGCR, and the results in Figure 6A are consistent with this interpretation. Are there other signs of unfinished replication, such as anaphase bridges or lagging chromosomes?

> This is an interesting question. Unfortunately, we do not have the answer. The study of all the consequences of *mcm3-2KR*, including incomplete DNA replication, is one of the many interesting directions suggested by our finding here. We appreciate the suggestion to search for such visual evidence and would look forward to this analysis in the future. Our current study focuses on the regulation of loaded MCM, which is disrupted in the *mcm3-2KR* mutant.

Other Points
5) Lines 12, 13: Duplicative use of support/supported.

> Corrected.

6) Lines 34 ff: This description is rather cryptic and could be elaborated.

> We have included the references reporting that SUMO targets many DNA replication proteins.

7) Line 86: The allele designation mcm3-29KR is used twice to denote different mutations. The nomenclature should be unique and defined.

> Clarified: *mcm3-29KR-1* has K767R and K768, while *mcm3-29KR-2* has K768R and K767.

8) Figure 1D: Nomenclature for 39KR with K767 or 768 restored is difficult to understand. Please change or explain better.

> Clarified.

9) The potential impact of the K767/768 sites on sumoylation on other residues should be discussed more.

> See Line-114. The effect of *mcm3-2KR* on sumoylation of the other MCM subunits, besides Mcm3 itself, is best explained by an indirect effect. The requirement of Cdc6 suggests that SUMO modifies the loaded MCM on chromosomes. Thus, a lower amount of loaded MCM means less MCM available for SUMO modification.

**Responses to Reviewer 2.**

I have one major point that the authors should clarify to support their conclusion that the SUMOylation of MCM3 K767 and K768 plays a role in the MCM loading. For phenotypic analyses in Figures 3 to 5, the authors used the MCM3 2KR mutant in which MCM3 K767,768R was expressed from the endogenous locus. I wonder if the MCM3 2KR forms the MCM2-7 complex efficiently as MCM3 WT. I understand that the mutant forms such complex when overexpressed for in vitro assay (Figure 4C). But this has not been tested in the MCM3 2KR mutant. If the MCM2-7 hexamer level was reduced in this mutant, it explains all phenotypes observed in Figures 3 to 5, which does not support the authors' proposal. Therefore, the authors should compare the level of the MCM2-7 complex between the MCM3 WT and 2KR cell lines.

> This is a good point. Indeed, MCM loading occurs in a series of steps that involve first the loading of MCM single hexamer, an intermediate, and then the loading of MCM double hexamer at replication origins in G1 cells. To test this, we performed co-IP experiment and found that *mcm3-2KR* reduces the amount of loaded MCM in G1 cells (new Figure 4A and Supplementary Figure 2). Importantly, *mcm3-2KR* has little effect on the association among MCM subunits in G2/M cells when loaded MCM is absent. However, this co-IP method does not distinguish single versus double hexamer whose relative amounts are unknown. For this reason, we used the dual tagging strategy and found that *mcm3-2KR* reduces the level of MCM double hexamer. Collectively, these results demonstrate that *mcm3-2KR* compromises the amount of loaded MCM in G1 cells, which may include single and double hexamers. The observed MCM association in G2/M cells is unregulated that is known to occur, and this is not affected by *mcm3-2KR*.

We speculate that *mcm3-2KR* could impair the loading of MCM single hexamer, the formation of double hexamer, and/or the stability of these MCM hexamers. Accordingly, we have revised the title and clarified the text about these possibilities.

Minor issues
Lines 117-118: "Thus, mcm3-2KR cells have an origin independent MCM-DH loading defect." What does it mean? Should it be "Thus, mcm3-2KR cells have an MCM loading defect at replication origins"?

> Corrected as: *mcm3-2KR* reduces the amounts of MCM loaded at both early and late origins. We removed the use of “loading” since our experiments measure the loaded MCM, not the loading process itself.

Supplementary Table 2: The genotype of HZY2337 and HZY1954 appears to be identical.

> Corrected: HZY2337 is used to analyze dGCR and is derived from HZY2131 (dGCR assay), while HZY1954 is used to analyze MCM sumoylation and is derived from HZY2101 (HF-SUMO).

**Responses to Reviewer 3.**

Major issues:

1. Line 113-118 and title: The authors suggest that the mcm3-2KR mutant impairs MCM loading at origins. It may equally be that sumoylation protects MCM against untimely unloading. The authors should provide experimental data to distinguish between these two models.

> We have previously discussed these models (loading versus unloading), which are not mutually exclusive. To avoid giving a false impression that we favor one over another, we have revised the text (Line 118) and changed the title accordingly. Although our finding raises the possibility of a putative MCM unloading activity, such an activity has not been established in the literature, despite an early clue (Cocker *et al*, 1996). While we share the curiosity of the reviewer about this possibility, proving the existence of a putative MCM unloading activity would open a new direction in the field of DNA replication, which in our opinion is beyond the scope of this study because this task requires a different approach.

2. Is it the CMGs that contain sumoylated MCMs that fire replication in S phase?

> We do not know much about CMG sumoylation and are not aware of any convincing evidence for a role of SUMO in CMG activation, published or unpublished. Our identification of K767/768 sumoylation of Mcm3 is the first example of site-specific MCM sumoylation being functionally important.

3. What fraction of MCM3 is sumoylated at K767/K768? The observation that the three major MCM3-SUMO bands can be removed independently by single mutations and no higher order species are observed suggest that MCM3 is primarily mono-sumoylated at only 1 lysine, which indicates that either sumoylation at the three sites are mutually exclusive or more likely that only a small fraction of MCM3 is sumoylated at any given time. Knowing the fraction of sumoylated Mcm3 is important because it would help distinguish if SUMO serves a constitutive function in MCM or a transient role e.g. during opening of the DNA gate in MCM to load MCM.

> The fraction of Mcm3 that is sumoylated at K767/768 in cells is low. For instance, we do not detect sumoylated Mcm3 in the purified double hexamer, unless we purify total sumoylated proteins to detect sumoylated Mcm3. Thus, sumoylation of MCM is sub-stoichiometric. This SUMO modification may transiently affect the loading or unloading of MCM through mechanisms yet to be discovered. Ultimately, addressing this question requires a complete reconstitution of SUMO-dependent MCM loading and/or unloading reactions, an important task for future studies.

4. Line 175 and elsewhere: There is good evidence that MCM is not loaded as a double hexamer, but rather that single hexamers are loaded sequentially to form the double hexamer (Miller et al, 2019). Therefore, the reduced level of MCM-DH likely reflects a reduction in DNA-bound MCM and therefore less opportunity for the MCM-DH to form. Please modify the text accordingly.

> We agree that both MCM single and double hexamers are present at DNA replication origins with single hexamer being an intermediate, although their relative amounts in cells are unknown. Because MCM double hexamer is the final product of MCM loading, we chose to analyze this species using the dual-tagging method, which distinguishes double hexamer from other MCM complexes. We have now included new co-IP result (new Figure 4A), showing that *mcm3-2KR* reduces the amount of loaded MCM in G1. Importantly, *mcm3-2KR* has little effect on the unregulated MCM complex formation in G2/M phase cells when loaded MCM is absent. Thus, this Mcm3-SUMO modification could regulate the loading of both single and double MCM hexamers, collectively referred as loaded MCM. We have clarified the text accordingly.

5. The authors examine MCM loading at an early and late ARS, but the notion that MCM3 sumoylation promotes genome-wide loading onto DNA would be strengthen, if the authors could demonstrate that mcm3-2KR locates to the soluble fraction and MCM3 to the chromatin fraction in WCE.

> In our hands, the conventional fractionation technique to separate chromatin from soluble fractions has been unreliable for the purpose of detecting ~ 2-fold reduction in various MCM complexes caused by *mcm3-2KR*, although the phenotypes of this mutant are dramatic. Because of this, we have chosen the more specific and accurate methods (ChIP-qPCR and co-IP) to evaluate various MCM complexes. The co-IP experiments show that *mcm3-2KR* reduces the overall level of loaded MCM (single and double hexamer, new Figure 4A and Supplementary Figure 2). However, we cannot exclude the possibility that the loaded MCM may be disproportionally reduced at different origins (see Lines 124-126). The use of ChIP-seq could be a better alternative, however, whether this method could reliably measure 2-fold changes is unknown. The soluble pool of each MCM protein in cells is in considerable excess (possibly >100 fold), so its amount is unlikely to be affected and thus uninformative.

Minor corrections

1. In the introduction on page 4, the impression is given that the regulation of replication by SUMO is ”not supported by direct evidence”, which does not give sufficient credit to the extensive work on PCNA sumoylation in both yeast and human.

> Extensive studies have shown that PCNA sumoylation acts to recruit the Srs2 helicase for DNA repair. Based on our knowledge, mutations eliminating this PCNA SUMO modification in yeast do not cause a noticeable effect on cell growth, chromosomal DNA replication, or the accumulation of GCRs. Therefore, it is inappropriate to assign a major role of PCNA sumoylation in chromosomal DNA replication or preventing GCRs here.

2. Figure 1D: the labels “K768R” are confusing because these cells are K768 WT, if I understand the figure correctly.

> Clarified in the figure legend. The top panel has single *mcm3-KR* mutants as indicated. The bottom panel shows the effects of restoring either K767 or K768 in the *mcm3-8KR* and *mcm3-39KR* backgrounds.

3. Line 157: change “Pulse-field gel electrophoresis” to “Pulsed-field gel electrophoresis”.

> Corrected.
